# Relation between fetal anthropometric parameters and cord blood adiponectin and high-sensitivity C-reactive protein in gestational diabetes mellitus

**DOI:** 10.1590/2359-3997000000235

**Published:** 2017-01-27

**Authors:** Mohammad Reza Aramesh, Masoud Dehdashtian, Arash Malekian, Shiva ShahAli, Kobra Shojaei

**Affiliations:** 1 Department of Pediatrics Ahvaz Jundishapur University of Medical Sciences Ahvaz IR Iran Department of Pediatrics, Division of Neonatology, Ahvaz Jundishapur University of Medical Sciences, Ahvaz, IR Iran; 2 Ahvaz Jundishapur University of Medical Sciences Ahvaz IR Iran Student Research Committee, Ahvaz Jundishapur University of Medical Sciences, Ahvaz, IR Iran; 3 Department of Obstetrics and Gynecology Ahvaz Jundishapur University of Medical Sciences Ahvaz IR Iran Department of Obstetrics and Gynecology, Ahvaz Jundishapur University of Medical Sciences, Ahvaz, IR Iran

## Abstract

**Objectives:**

The objectives were to evaluate the relation between fetal anthropometric parameters and cord blood concentration of adiponectin and high sensitivity C-reactive protein (hs-CRP). Subjects and methods: A total of 104 pregnant women (52 with gestational diabetes mellitus [GDM], 52 with normal glucose tolerance (NGT) participated. Venous cord blood samples were obtained at delivery, centrifuged and the plasma was stored at -20°C. The samples were assessed for adiponectin and hs-CRP using the ELISA method. Statistical analysis was done using SPSS software.

**Results:**

The adiponectin concentration was higher in the GDM group than in the NGT group (11.05 ± 4.1 µg/mL in GDM vs. 5.34 ± 2.63 µg/mL in NGT, p < 0.001). GDM was also higher in neonates delivered at later gestational ages (p < 0.001, Pearson correlation = 0.59). There was a positive correlation between cord blood adiponectin and birth weight in the GDM group (p < 0.001, Pearson correlation = 0.619) but not in the NGT group. There was no significant correlation between adiponectin and infant length or head circumference. There was also no significant difference in cord blood hs-CRP concentration between groups. No relation was found between hs-CRP and newborn anthropometric parameters.

**Conclusion:**

In the GDM group, adiponectin concentration was considerably higher and had a positive correlation with the ponderal index and birth weight which was not found in the NGT group.

## INTRODUCTION

Gestational diabetes mellitus (GDM) is impaired glucose tolerance that develops or is first diagnosed during pregnancy ( 1 - 3 ). Recent studies have shown a 20-fold increase in the prevalence of GDM. A further 4-fold increase in prevalence is anticipated ( 3 , 4 ) after adoption of the new diagnostic criteria suggested by the International Association of Diabetes and Pregnancy Study Groups (IADPSG) ( 1 , 5 ). The presence of GDM means that many infants are exposed to hyperglycemic conditions in utero which can cause neonatal adiposity and metabolic disorders later in life.

The pathogenesis of GDM and its features that result in obesity and metabolic disorders remain unclear. Recent studies have shown that the inflammatory system may play a role in the development and pathogenesis of GDM ( 1 ). Insulin resistance and inflammation are major features of maternal metabolic state in women with GDM. Both of these conditions can affect fetal growth ( 2 ). The inflammatory environment alters placental gene transcription and fetal metabolic programming. Genes for lipid metabolism and those for inflammatory pathways are upregulated in the placenta of women with GDM ( 2 ). This upregulation increases or unbalances production of inflammatory cytokines and energy metabolism regulatory cytokines such as adipokines ( 2 , 6 , 7 ), which increases adiposity at birth ( 8 , 9 ) and predisposes the newborn to become overweight and develop metabolic diseases such as impaired glucose tolerance, metabolic syndrome, and cardiovascular disease ( 2 , 6 - 8 ). Previous studies have focused on the pathophysiologic pathways and cytokine levels in GDM and their possible effect on offspring comorbidities later in life ( 1 , 6 , 7 , 10 ).

Adiponectin is a cytokine that is released exclusively by adipocytes in adults ( 9 , 11 - 13 ). Maternal adiponectin has a high molecular weight; therefore, it probably does not pass through the placental barrier; its concentration in the umbilical cord is of fetal origin ( 14 , 15 ). The origin of adiponectin in the fetus is not clearly understood ( 13 ). In adults, adiponectin concentration is inversely related to adiposity, but its association with adiposity is poorly understood ( 9 , 11 , 13 ). Some studies indicate a positive correlation between cord plasma adiponectin concentration and adiposity in infants ( 8 , 12 , 14 , 16 - 20 ) and some have found no significant correlation ( 9 , 15 ).

C-reactive protein (CRP) is an acute phase protein in plasma synthesized by the liver in response to pre-inflammatory cytokines. Its concentration increases 12-24 hours after the commencement of an inflammatory process and preserves its level throughout the inflammation. There is a positive correlation between CRP concentration and insulin resistance and elevated levels of CRP have been reported in GDM. A growing body of evidence suggests that CRP is related to development and progression of cardiovascular disease ( 21 ).

The present study evaluated the relationship between fetal anthropometric parameters, cord blood adiponectin and hs-CRP concentration at birth and compared mean cord plasma adiponectin and hs-CRP concentrations in the GDM and normal glucose tolerant (NGT) groups.

## SUBJECTS AND METHODS

This analytical, case-controlled study was conducted at Imam Khomeini Hospital (a university-affiliated hospital) in the city of Ahvaz in Iran. Recruitment began in June 2014 and ended in July 2015. The study protocol was approved by the Research Ethics Committee of Ahvaz Jundishapur University of Medical Sciences. Informed consent was obtained from all patients. Prior to participation, all the participants underwent an oral glucose tolerance test (3 hour, 100 g glucose). Those who had two or more values exceeding the thresholds of the GDM diagnostic criteria as suggested by the National Diabetes Data Group (NDDG) were included in the GDM group. Women who had no values meeting a NDDG criteria were included in the NGT group. Those who had only one value exceeding the threshold of the criteria were excluded from the study ( 22 ).

Inclusion criteria were a gestational age at delivery of 35 to 41 weeks, singleton pregnancy, first minute Apgar score > 7 and uncomplicated delivery. Exclusion criteria were substance abuse by mother (including cigarettes, alcohol, etc.), chronic disease and overt diabetes in the mother and the presence of congenital anomalies.

Eventually 104 pregnant women entered the study (52 women in the GDM group and 52 in the NGT group). Of the 52 women with GDM, 38 women were given a 40% carbohydrate diet to control their GDM and 14 women were treated with insulin.

### Clinical and demographic data

The demographic data was gathered by questionnaire. The height and weight of the pregnant women were measured using a calibrated medical scale and recorded. The body mass index (BMI) was calculated as BMI = weight (kg)/height (m^2^). The length of the newborns was measured using a calibrated length board and their weight with a calibrated scale. To assess fetal growth pattern, the ponderal index (PI) was calculated as



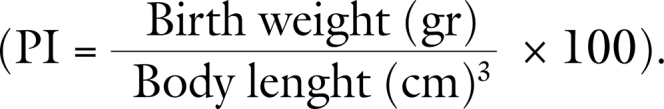



### Lab measurement

Venous cord blood samples were obtained from the 104 full-term healthy infants. After delivery and cord clamping, 5 mL of cord blood was collected from the umbilical vein in prepared and heparinized tubes at ambient temperature using the aseptic method. The samples were centrifuged and the plasma was kept frozen and stored at -20^o^C until analysis.

Adiponectin was assayed using an ELISA kit specific to human adiponectin (Biovendor; Laboratorni Medicina; Czech Republic). Serum adiponectin was measured as micrograms per milliliter (µg/mL). Quantitative high sensitivity C-reactive protein (hs-CRP) was assayed using an i-CHROMA kit for fluorescence immunoassay specifically for determination of human hs-CRP (Boditech Med Europe; United Kingdom). Serum hs-CRP was measured as milligram per milliliter (mg/mL).

### Statistics

The data were analyzed by SPSS 13 software (SPSS; USA). The unpaired student T-test was utilized to assess differences between groups. Simple linear regression was employed to eliminate the influence of other predictor values. Pearson correlation was used to analyze the bivariate correlation between adiponectin levels and anthropometric parameters.

## RESULTS

All infants were healthy and were born at Imam Khomeini Hospital. The demographic and clinical data of the study population are shown in Table 1 . Although there was no statistically significant difference in age, parity and BMI between groups. The offspring of the GDM group had higher adiposity, were more prone to be delivered by cesarean section, and were delivered one week earlier on average than the NGT group ( Table 1 ).

**Table 1:** Demographic and clinical data of the study population

	Whole group	NGT group	GDM group	P value
n	104	52	52	
Maternal characteristics:				
Age (years)	27.89 ± 5.63 (17-40)	26.8 ± 5.05	28.9 ± 6.02	NS
BMI	25.17 ± 4.89	24.96 ± 5.24	25.37 ± 4.53	NS
Parity				
0	41	19	22	NS
1	37	21	16	
> 1	26	12	14	
Children (boys/girls)	51/53	27/25	24/28	NS
Gestational age at delivery (weeks)	38.12 ± 1.46 (35.44-41)	38.65 ± 1.45	37.6 ± 1.28	< 0.001
HbA1c	-	-	5.24 ± 0.32	
Delivery				< 0.001
Vaginal	93	50	43	
Caesarian	11	2	9	
Neonatal characteristics:				
Birth weight (kg)	3.28 ± 0.45 (2.30-4.15)	3.04 ± 0.35	3.53 ± 0.40	< 0.001
Ponderal index (gr⁄cm^3^)	2.57 ± 0.29 (2.04-3.39)	2.40 ± 0.18	2.74 ± 0.28	< 0.001
Birth length (cm)	50.32 ± 1.72 (47-56)	50.14 ± 1.43	50.50 ± 1.96	NS
Head circumference (cm)	34.17 ± 0.72 (30-36)	34.12 ± 0.79	34.22 ± 0.65	NS
Cord serum adiponectin (µg/mL)	8.20 ± 4.470 (0.8-22.30)	5.34 ± 2.63	11.05 ± 4.1	< 0.001
Cord serum hsCRP (mg/mL)	0.19 ± 0.30 (0.1-2.0)	0.19 ± 0.28	0.19 ± 0.33	NS

Data are mean ± SD; HbA1C: glycosylated hemoglobin; NS: not significant.

All of the 104 infants were included in the study (51 males and 53 females; gestational age: 35-41 weeks; birth weight: 2300-4150 g). No significant differences were observed between groups. The mean birth weight and PI were higher in the GDM group than the NGT group (birth weight: 3535.1 ± 400 g vs. 3041.7 ± 350 g, PI: 2.74 ± 0.28 g/cm^3^ vs. 2.40 ± 0.18 g/cm^3^; p < 0.001). There was no significant difference between the mean length (50.50 ± 1.96 cm in GDM group vs. 50.14 ± 1.43 cm in NGT group; p = 0.29) and head circumference (34.22 ± 0.65 cm in GDM group vs. 34.12 ± 0.79 cm in NGT group, p = 0.5) between groups.

The adiponectin present in the cord blood ranged from 0.8 to 22.30 µg/mL. Mean cord blood adiponectin was 11.05 ± 4.1 µg/mL in the GDM group and 5.34 ± 2.63 µg/mL in the NGT group and was significantly higher in the GDM group (p < 0.001). In addition, there was a significant correlation between cord blood adiponectin and birth weight in the GDM group (p < 0.001, Pearson correlation = 0.619). No such relation was found in the NGT group. There was no significant correlation between adiponectin and length or head circumference of the infants.

No difference in adiponectin concentrations was observed according to gender or by mode of delivery between groups. Cord blood adiponectin concentrations were higher at later gestational ages and there was a significant correlation between gestational age and adiponectin concentration (p < 0.00; Pearson correlation = 0.59). There was no significant difference in cord blood hs-CRP concentration between groups. No significant relationship was found between hs-CRP and newborn anthropometric parameters.

## DISCUSSION

Adiponectin is a cytokine of great importance because it can cause establishment of metabolic diseases. Adiponectin concentration has a reverse correlation with inflammatory diseases such as obesity and cardiovascular disease ( 23 , 24 ). Studies have shown that adiponectin affects insulin sensitivity, β oxidation and inflammatory pathways. The fact that adiponectin concentrations increase at later gestational ages suggests a role for it in the early growth and development of the fetus ( 9 , 13 ). The present study revealed that the mean cord blood adiponectin concentration in the GDM group was significantly higher than that in the NGT group. Previous studies have reported lower cord blood adiponectin concentrations in the GDM group ( 11 ).

The data showed a significant correlation between PI (Pearson correlation: 0.421), birth weight (Pearson correlation: 0.619) and cord blood adiponectin concentration. Increased adiponectin levels may play a role in increased birth weight and PI. Ballesteros and cols. ( 11 ) reported similar findings. In contrast, Lindsay and cols. ( 9 ) found no significant correlation between adiponectin concentration and anthropometric parameters. Another study suggested a reverse correlation between PI and adiponectin concentration ( 11 , 25 ). Some studies have shown a positive correlation between adiponectin concentration and birth weight ( 8 , 12 , 14 , 16 - 20 ), but few have failed to find a significant correlation ( 9 , 15 ). The current study found a positive correlation between adiponectin concentration and gestational age in the GDM group although there was no such correlation in the NGT group. Ballesteros and cols. ( 11 ) reported similar results, but Lindsay and cols. ( 9 ) found no significant correlation between gestational age and adiponectin.

There was no significant correlation found for adiponectin concentrations versus delivery mode, which agrees well with the findings of Lindsay and cols. ( 9 ). The results also show no difference in adiponectin levels by gender. By contrast, Lindsay and cols. ( 9 ) reported a difference by gender for adiponectin concentration. No difference in adiponectin concentration was found between women with GDM who were treated with a 40% carbohydrate diet and those who were treated with insulin in the present study.

This study found no significant difference in cord blood hs-CRP concentration between groups. These findings were similar to those of Mordwinkin and cols. ( 26 ) and Jahromi and cols. ( 27 ). No significant relation was found between hs-CRP and newborn anthropometric parameters in the present study. To our knowledge the present study is one of the first to investigate the relation between hs-CRP and newborn anthropometric parameters. No similar studies were found to compare with the results of the present study.

The study had certain limitations. Only Iranian women were involved, so the results cannot be extrapolated to other ethnicities. Moreover, financial limitations prevented adoption of IADSPG criteria for screening and diagnosing GDM. This study is one of few to assess the level of adiponectin and hs-CRP in GDM and their relation with fetal anthropometric parameters. Inflammatory pathophysiology may play a role in the development of GDM ( 1 ), but the mechanism is not fully understood. The findings of the previous studies have conflicted. Further investigation is needed to examine whether or not a significant difference exists between cord serum concentration of adiponectin and hs-CRP among women with GDM compared to healthy pregnant controls and investigate their relation to fetal anthropometric parameters. To summarize, a significantly higher adiponectin concentration was found in the GDM group and had a positive correlation with PI and birth weight which was not observed in the NGT group.
